# Generation of new rice germplasms with low amylose content by CRISPR/CAS9-targeted mutagenesis of the *FLOURY ENDOSPERM 2* gene

**DOI:** 10.3389/fpls.2023.1138523

**Published:** 2023-03-13

**Authors:** Xiaohong Song, Zhihui Chen, Xi Du, Bin Li, Yunyan Fei, Yajun Tao, Fangquan Wang, Yang Xu, Wenqi Li, Jun Wang, Guohua Liang, Yong Zhou, Xiaoli Tan, Yulong Li, Jie Yang

**Affiliations:** ^1^ School of Life Science, Jiangsu University, Zhenjiang, Jiangsu, China; ^2^ Institute of Food Crops, Jiangsu Academy of Agricultural Sciences/Key Laboratory of Germplasm Innovation in Downstream of Huaihe River Ministry of Agriculture and Rural Affairs, Nanjing, Jiangsu, China; ^3^ Jiangsu Co-Innovation Center for Modern Production Technology of Grain Crops, Yangzhou University, Yangzhou, China

**Keywords:** *FLOURY ENDOSPERM 2*, amylose content (AC), eating and cooking quality (ECQ), japonica rice, CRISPR/Cas9, genome editing

## Abstract

*FLOURY ENDOSPERM 2* (*FLO2*), encoding a tetratricopeptide repeat domain (TPR)-containing protein located in the nucleus, is considered to be a regulatory protein that controls the biosynthesis of seed storage substances. The diversity of *flo2* allele is attributable for the variations in grain appearance, amylose content (AC), and physicochemical properties, influencing the eating and cooking quality (ECQ) of rice. In this study, we used CRISPR/Cas9 to introduce loss-of-function mutations into the *FLOURY ENDOSPERM 2* gene in Suken118 (SK118), a widely cultivated elite *japonica* rice variety in Jiangsu, China. Physiochemical analyses of the *flo2* mutants were congruent with previous studies, exhibiting lowered AC and viscosity, risen gel consistency (GC) and gelatinization temperature (GT) values, which were all instrumental to the improvement of ECQ. However, the wrinkled opaque appearance and the decrease in grain width, grain thickness and grain weight imply trade-offs in grain yield. Despite the *ex-ante* estimation for low yielding, the superior ECQ in these novel genotypes generated by using genome editing approach may have the potential for formulating high value specialty food.

## Introduction

Rice (*Oryza sativa* L.), one of the most important crops worldwide, provides staple food for feeding half of the world’s population. The steady increase in grain yield, coupled with the rapid improvements in human living conditions, has fostered ever increasing public demands for foods of high quality and high versatility (Concepcion et al., 2015; Huo et al., 2017). Consequently, the rice quality, as measured by a range of standardized parameters, including milling, appearance, nutrition and eating and cooking quality (ECQ), has been commonly used to select for and evaluate the breeding lines prior to commercial release (Zhang et al., 2018; Huang X. et al., 2021; Hu et al., 2021; Zhang H. et al., 2021). Amylose and amylopectin constitute starch that is the dominant component in rice grains and accounts for about 90% of the weight of the rice endosperm (Zhou et al., 2002; Wang et al., 2018). As the most important quality trait, ECQ is affected by multiple factors such as amylose content (AC) and an eclectic list of physicochemical properties of endosperm (Lu et al., 2013; Chun et al., 2015; Van Hung et al., 2016). Despite the recent increase in the awareness of the health benefits of high AC rice, it appears that a seemingly polarized market has emerged, especially for the formulation of specialty food, where the low AC trait is desired as it could well be attributed for the favorable food texture and taste (Sagare et al., 2020; Hu et al., 2021). Further, some rice cultivars, such as Nanjing 46 and Nanjing 9108, with relatively high ECQ and low AC (10-15%) are popular with consumers in China and the broad East Asian region, which are soft yet not sticky after cooking, and are defined as soft japonica rice (Li et al., 2018; Hu et al., 2021; Zhang C. et al., 2021). Therefore, there is impetus to develop rice genotypes with low AC to meet market requirements.

*FLOURY ENDOSPERM 2* (*FLO2*), a member of a conserved gene family in plants, encodes a tetratricopeptide repeat domain (TPR)-containing protein located in the nucleus (She et al., 2010; Wu et al., 2015; Kolli et al., 2019; Suzuki et al., 2020). It has been reported that *FLOURY ENDOSPERM 2* could not only modulate the expression of starch synthesis-related genes including *OsAGPL2, OsAGPS2b, OsGBSSI, OsBEI, OsBEIIb, OsISA1* and *OsPUL*, but also directly impact on the amylose biosynthesis by manipulating the splicing efficiency of *Wx* pre-mRNA (Wu et al., 2015; Cai et al., 2022; Feng et al., 2022; Sreenivasulu et al., 2022). Previous studies have shown that *flo2*, the loss-of-function mutant of *FLOURY ENDOSPERM 2*, produced two distinct phenotypes, one with white and floury endosperm, and the other with dull grain (She et al., 2010; Hunt et al., 2013; Boehlein et al., 2018). In the former, starch granules were small, spherical and loosely packed with large air space (Malinova et al., 2017; Jiang et al., 2018; Suzuki et al., 2020). The *FLOURY ENDOSPERM 2* also affected grain size as demonstrated by both overexpression and null mutation approaches (She et al., 2010; Zhang et al., 2013). In addition, *flo2* mutant was also endowed with alterations in grain physicochemical properties including grain breakdown, setback and consistency (Wu et al., 2015; Zhang et al., 2017). Therefore, *FLOURY ENDOSPERM 2* plays an important role in the determination of grain quality by regulating the accumulation of storage substances in the endosperm. Previous methods to generate *flo2* mutants by chemical mutagens such as EMS have the disadvantages of safety risk, low efficiency and uncontrollable direction (Wu et al., 2015; Kihira et al., 2017; Wu et al., 2019).

In the last few years, as one of the most advanced systems for genome editing, CRISPR/Cas has been commonly used to make precise and predictable genome modifications in crop plants to obtain desired traits (Zhu et al., 2020; Gao, 2021). This is especially true for rice, where impressive progress has been made and a plethora of traits have been modified by applying the CRISPR/Cas9 editing system, by virtue of rice’s economic significance and the ease of transgenic regeneration (Zhou et al., 2014; Ma et al., 2021). For grain quality improvement, *Wx* is a key gene that encodes granule binding starch synthase I (GBSSI) involved in amylose biosynthesis. It has been genetically edited to manipulate AC and/or improve ECQ in a number of recent studies in rice (V.M. Butardo et al., 2017; Bello et al., 2019; Fei et al., 2019; Huang et al., 2020; Zeng et al., 2020; Huang L. et al., 2021; Xu et al., 2021). Likewise, a number of other key genes involved in amylopectin fine structure, such as *SSII-2, SBEIIb, BEIIb* and *ISA1*, have been edited for ECQ improvements (Crofts et al., 2015; Sun et al., 2017; Chao et al., 2019; Tappiban et al., 2022).

In this study, to induce AC and improve ECQ of a widely cultivated elite japonica variety, Suken118 (SK118), two loss-of-function mutants of *FLOURY ENDOSPERM 2* gene were generated by CRISPR/Cas9 technology. Our results show that knockout of *FLOURY ENDOSPERM 2* gene can significantly reduce the content of straight chain powder. The physicochemical properties of *flo2* mutants showed that lower AC and viscosity, higher gelation degree and gelatinization temperature were helpful to improve the edible and cooking quality of rice. Therefore, *FLOURY ENDOSPERM 2* has potential application value in green and healthy rice breeding.

## Results

### Generation and identification of *flo2* mutants in rice

**Figure 1:**
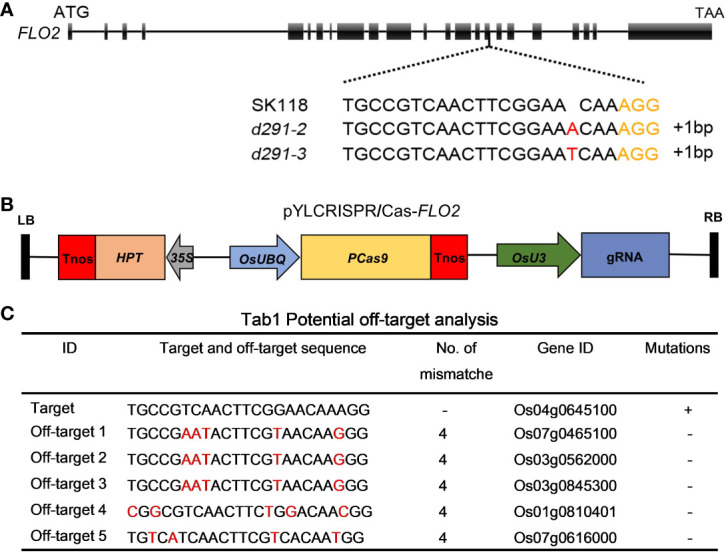
CRISPR/Cas9-induced mutations in the *FLOURY ENDOSPERM 2* genes. **(A)** Schematic of the *FLOURY ENDOSPERM 2* gene structures and target sites. Exons and introns are indicated with black rectangles and black lines, respectively. The spacer and PAM sequences were marked in black and orange. **(B)** The expression vector of pYLCRISPR/Cas9-*Flo2*. The expression of Cas9 is driven by the maize ubiquitin promoter (*OsUBQ*); the expression of the sgRNA scaffold is driven by the rice *OsU3* small nuclear RNA promoter; the expression of hygromycin (*HPT*) is driven by CaMV35S promoter. Tnos, the terminator; LB and RB, left border and right border, respectively. **(C)** Potential miss analysis. Red fonts indicate different bases.

**Figure 2:**
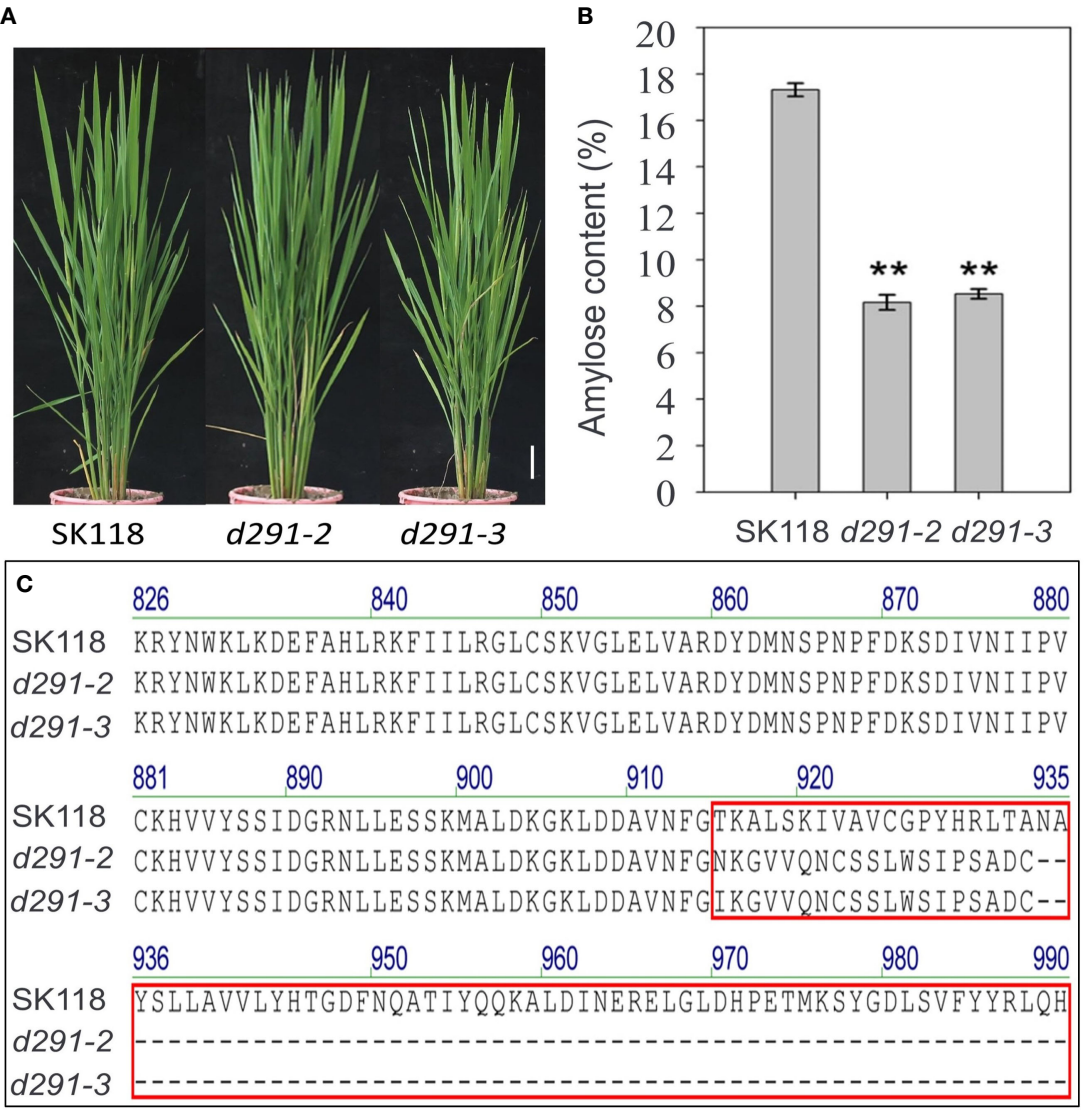
Phenotype and amino acid variations assays. **(A)** Phenotypes of *flo2* mutant lines (*d291-2* and *d291-3*) in SK118 background. Bar = 5 cm. **(B)** Amylose content (%). Data are given as the mean ± S.D. (n ≥ 3). Student’s t test: *P < 0.05; **P < 0.01; ns, no significance. **(C)** Amino acid variations of the FLO2 protein in the *flo2* mutant. Wild-type, SK118. Homozygous mutations identified at the target sites of *d291-2* and *d291-3* mutant lines in the T1 generation. Amino acid variations were indicated in red box, with the number representing the order in the proteins. The ellipsis dots represent premature stop codons.

To generate *flo2* mutants with the expectation to produce null mutations, sgRNA was designed in the coding region of *FLOURY ENDOSPERM 2*. The constructed expression vector *flo2* gRNA was expressed and driven by the *OsU3* promoter, and the Cas9 cassette was driven by the ubiquitin promoter (**Figure 1A**). We took advantage of these CRISPR/Cas expression vectors to transform rice variety Sk118 by Agrobacterium tumefaciens infecting rice calli (**Figure 1B**). Positive T0 transgenic plants were identified by PCR amplification of a fragment of the Hyg gene that was used as the selection marker. The target genomic region of *FLOURY ENDOSPERM 2* was amplified by a pair of primers (*FLO2*-TF/TR; **Table S1**) flanking the target site and sequenced. In addition, we failed to find any mutations in any of the potential off-target sites (**Figure 1C**). The sequencing results of T0 plants showed that the mutation rate was as high as 74.19%, among which the percentages of mutation by inserting the base “T” and “A” were the highest, being 30.43% and 19.57%, respectively. Two homozygous T1 mutant lines free of T-DNAs, named *d29-2* and *d29-3*, were selected for further research, featuring the insertion of a single base of “T” and “A”, respectively, resulting in frame shift and premature termination of translation (**Figure 2C**).

### Knockout of *FLOURY ENDOSPERM 2* gene has multiple effects on agronomic traits

**Figure 3:**
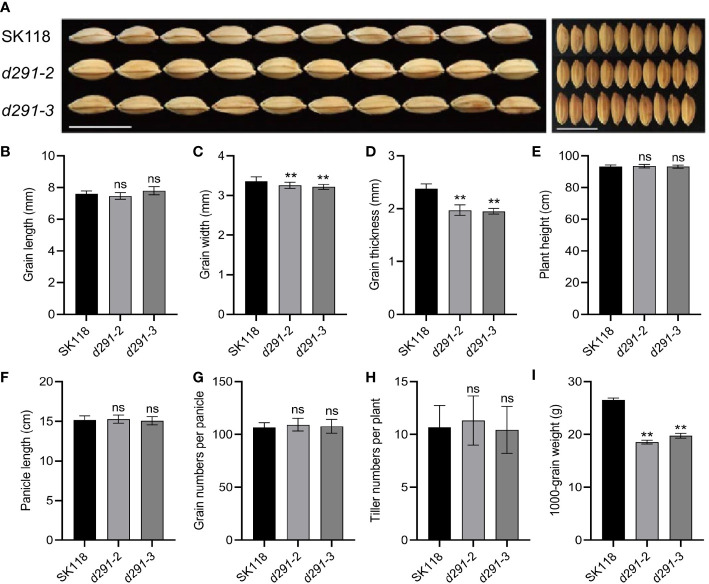
Knockout of *FLOURY ENDOSPERM 2* gene has multiple effects on agronomic traits. **(A)** Grain phenotypes of *flo2* mutant lines (*d291-2* and *d291-3*) in SK118 background. Bar = 1 cm. **(B)** grain length (mm). **(C)** grain width (mm). **(D)** grain thickness (mm). **(E)** plant height(cm). **(F)** panicle length (cm). **(G)** grain numbers per panicle. **(H)** tiller numbers per plant. **(I)** 1000-grain weight **(G)**. Data are given as the mean ± S.D. (n ≥ 20). Student’s t test: **P < 0.01; ns, not significance.

All the plants were grown in the experimental field in Jiangsu Academy of Agricultural Sciences, Nanjing, China, during normal rice-growing seasons. The edited plants derived from *d29-2* and *d29-3* did not exhibit discernible variations from wild type (WT) control in grain length, plant height, the panicle length, the number of grains per panicle and tiller numbers per plant (**Figure 2A**, **Figures 3A, B, E–H**). In contrast, the grain width and grain thickness of the *flo2* mutants were significantly lesser than WT control, which is congruent with the previous studies on chemically induced *flo2* mutants (She et al., 2010); (**Figures 3C, D**). Conceivably, the 1000-grain weights of *d29-2* and *d29-3* were reduced by 26.50% and 20.90%, respectively (**Figure 3I**).

Rice yield and grain appearance quality are complex quantitative characters, which are influenced by genetic background and environmental factors. Although *FLOURY ENDOSPERM 2* gene knockout mutants have multiple negative effects on grain appearance and yield, rice with low amylose content and high cooking quality has a huge market demand. In future studies, *FLOURY ENDOSPERM 2* gene and yield trait genes can be combined, and the mutual balance between yield and trait can be realized by using the interaction effect between different genes.

### Improvement of ECQ and taste value of *flo2* transgenic rice

**Figure 4:**
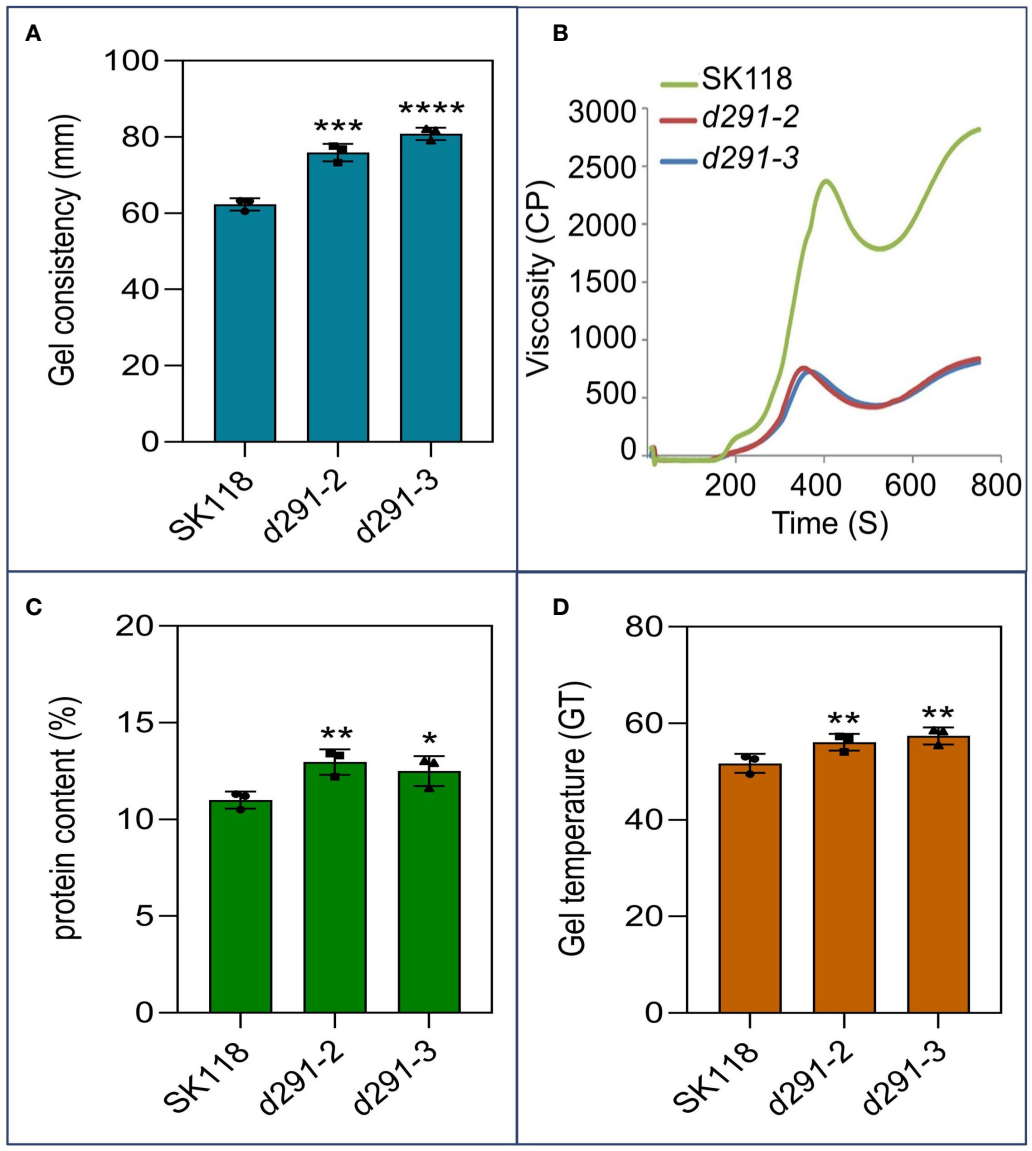
Improvement of ECQ and taste value of *flo2* transgenic rice. **(A)** Gel consistency (mm). **(B)** RVA spectra of rice flours. **(C)** Total protein content of the rice flours (%). **(D)** Gelatinization temperature (GT) of rice flours from SK118, *d291-2* and *d291-3*. Data are given as the mean ± S.D. (n ≥ 3). Student’s t test: *P < 0.05; **P < 0.01; ***P < 0.001; ****P < 0.0001.

To further assess the effects of *flo2* mutants on rice ECQ, we performed a number of assays on the grain. The gel consistency (GC) in *d29-2* and *d29-3* increased significantly relative to WT by 11.6 mm and 18.0 mm, respectively (**Figure 4A**). The gelatinization temperature (GT) of the mutant lines was about 22°C higher than WT (**Figure 4D**). The increases in GC and GT in *flo2* mutant relative to WT are favorable for improving ECQ in rice (Zhang et al., 2020; Sreenivasulu et al., 2022). The RVA pasting profiles of the *flo2* mutants were also distinct from WT, specifically, the peak paste viscosity (PKV), hot paste viscosity (HPV) and cold paste viscosity (CPV) of the mutants were significantly lower than those of WT (**Figure 4B**). The protein content (PC) was only increased of 1.68% and 1.64% in *d29-2* and *d29-3* mutants, respectively (**Figure 4C**), which had no significant effect on ECQ (Sreenivasulu et al., 2022). Generally, the increase of gel consistency is conducive to improving the taste quality of rice. Taken together, these results manifest the complex changes in the physicochemical properties of cooked rice grains in the *flo2* mutants, which are generally conducive to ECQ improvements.

Rice starch with a higher amylose content always shows faster retrogradation, leading to higher setback viscosity (SBV) of the starch, while starch with a lower amylose content has a higher relative crystallinity and short-range molecular order, resulting in higher swelling power, PKV and breakdown viscosity (BDV) (Wang et al., 2017; Cai et al., 2022). Previous studies showed that AC was positively correlated with PKV, HPV, CPV in a significant manner, whereas it was negatively correlated with adhesiveness, cohesiveness, and GT (Kong et al., 2015). Our results in this study on *flo2* lines that were endowed with low AC was concurrent with the reductions in these RVA pasting values, corroborating the previous reports. GC is an important indicator for distinguishing rice grains that do not undergo retrogradation (i.e., rice remains soft after cooking and cooling) (Zhang et al., 2020; Sreenivasulu et al., 2022). GT is also a crucial factor that affects rice ECQ. It is generally recognized that the rice grains with high GT would need more time to cook, and the texture of the cooked rice tends to be less sticky, especially when the cooked rice is cooled (Li et al., 2021; Sreenivasulu et al., 2022). The enhancements in GC and GT values in the *flo2* mutants, as unraveled in this study, are clearly favorable for ECQ amelioration.

### Opaque appearance phenotype of *flo2* mutants rice grain

**Figure 5:**
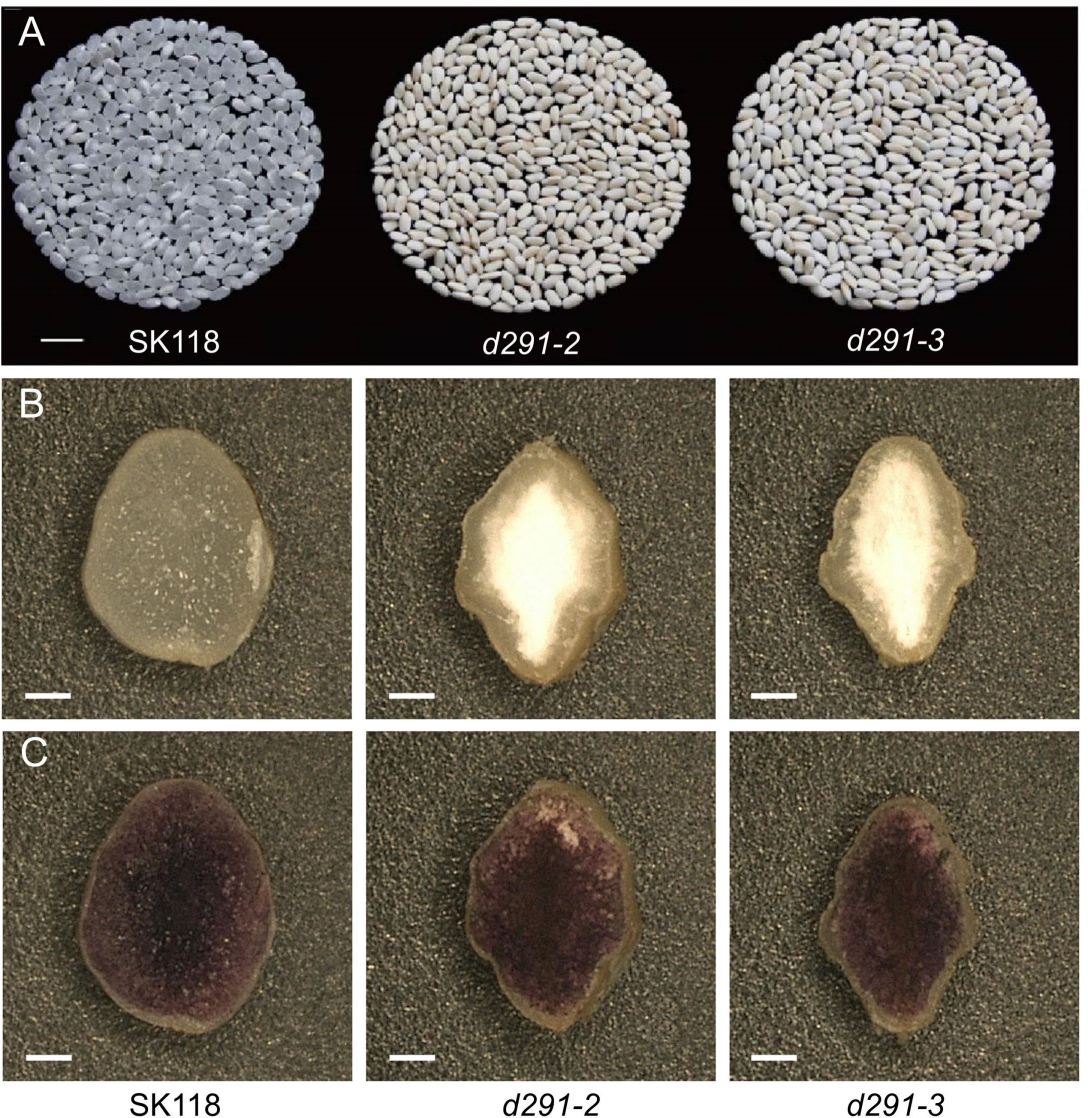
Appearance phenotype analysis of *flo2* mutants rice grain. **(A)** Appearance of polished rice from *flo2* mutant lines (*d291-2* and *d291-3*) in SK118 background. Bar = 1 cm. **(B)** Transverse sections of rice grains. Bar = 300 μm. **(C)** Rice grains stained with KI-I2. Bar = 300 μm.

The polished rice grains derived from *d29-2* and *d29-3* were featured with distinctive opaque and shriveled appearance (**Figure 5A**). There might be some phenotypic variations in grain appearance between the *FLO*-edited lines and the *flo2* mutants that were reported in previous studies, which could well be attributed to the different genetic backgrounds and cultivation practices used between different studies (She et al., 2010; Wu et al., 2015). The images of transverse sections showed that the endosperm of the *flo2* mutants were more opaque and powdery compared to WT at the center of the endosperm, but such differences were diminished away from the center region (**Figure 5B**).

We speculate that this is mainly due to the fact that there are many gaps between the poorly filled starch bodies in the endosperm. When the light shines on the endosperm in the internal area, the light diffuses between the gaps, causing the endosperm to appear powdery. When the inner amyloplasts are closely arranged and there is no gap, the light can directly pass through and present transparent endosperm. Such spatial variation in endosperm between *flo2* and WT is intriguing and warrants further investigation.

### Genome editing of *FLOURY ENDOSPERM 2* decreases amylose content significantly

Amylose has high binding capacity with iodine. The higher the AC is, the deeper the staining in the starch-iodine reaction becomes (Agasimani et al., 2013). As expected, the iodine staining of the endosperms in the transverse sections of *flo2* seeds were overtly lighter than WT, indicative of AC reduction in the mutants (**Figure 5C**). The measurement of AC by using AA3 continuous flow analyzer showed that the AC of *d29-2* and *d29-3* were 8.20% and 8.56%, respectively, which are in sharp contrast to that of WT (17.35%) (**Figure 2B**). Rather, AC in the *flo2* mutants resemble that of a typical soft rice (Wu et al., 2015). Starch is the first kind of storage material in rice. The reduction of 1000 grain weight of *flo2* mutants is mainly caused by the reduction of total starch content in endosperm of seeds. Furthermore, we detected the transcript levels of *FLOURY ENDOSPERM 2* in the WT (SK118), *d291-2* and *d291-3* plants. The transcript levels of *FLOURY ENDOSPERM 2* were decreased in the *d291-2* and *d291-3* plants, consistent with the previous study (**Figure S1**) (She et al., 2010).

### Morphology of starch granules in endosperm cells of *flo2* mutants

**Figure 6:**
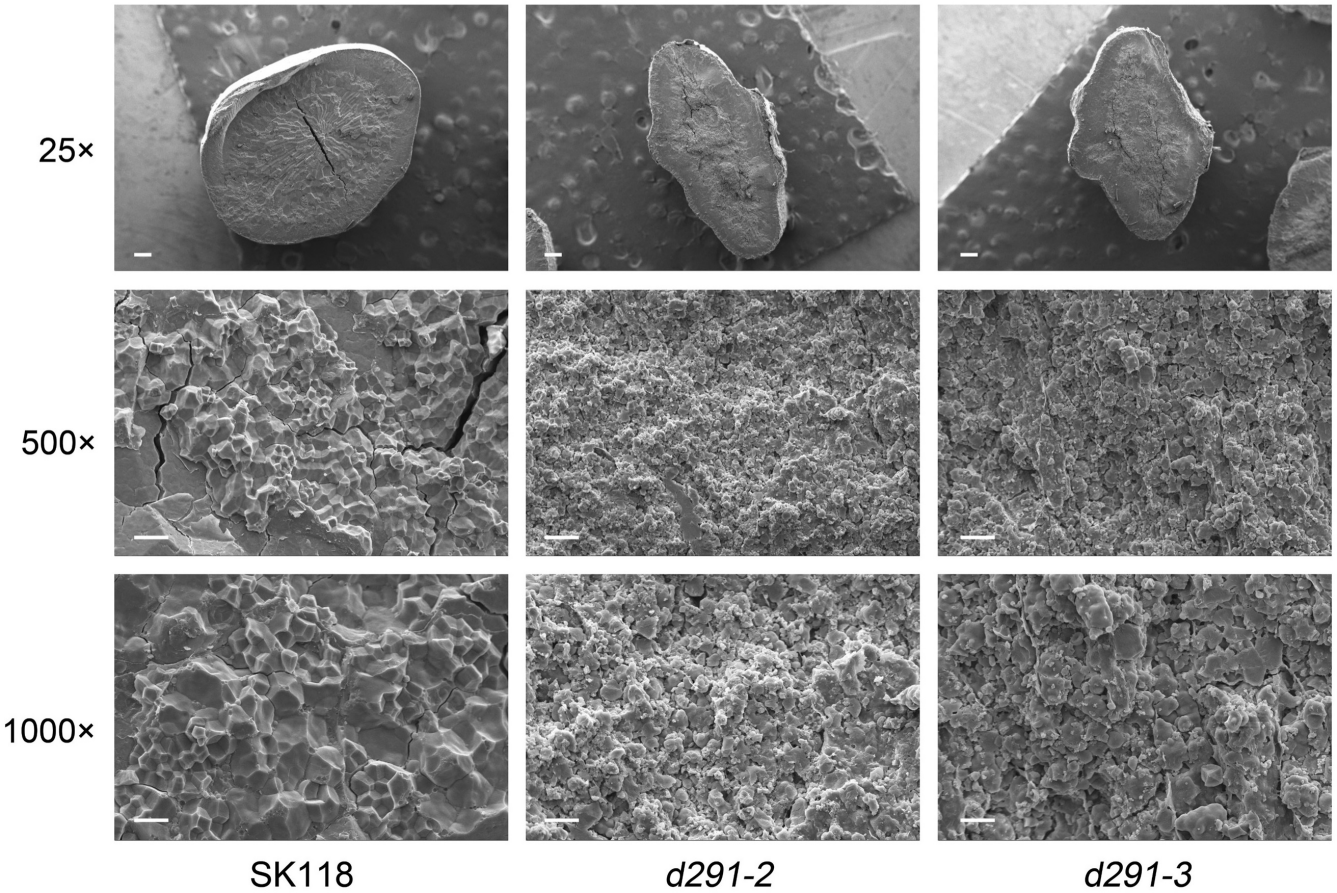
Morphology of starch granules in endosperm cells of *flo2* mutants. **(A)** Scanning electron microscopy images of the transverse section of *flo2* mutants and wild-type. 25×, Bars = 200μm. 500×, Bars = 20 μm. 1000×, Bars = 10 μm.

To explore the causes of endosperm changes, we observed the endosperm of wild type seeds and two mutant seeds with scanning electron microscopy. Further, scanning electron microscopy images showed that the starch grains of the *flo2* mutants were irregularly spherical and loosely arranged, contrary to the regular spherical shape and uniformity in size, and tight arrangement (**Figure 6A**). The loose arrangement of *flo2* mutants starch granules is the main reason for its opaque appearance.

We speculated that the starch accumulation in *flo2* mutants was poor, which led to the loose endosperm structure. During the ripening process of rice, the powder region of endosperm is easy to be broken under external pressure such as water loss, forming sub grains. A large number of cavities were left between loose starch grains, which led to diffuse reflection of light to form a silty, opaque endosperm phenotype.

## Discussion

In summary, in this study we have generated low AC/high ECQ rice genotypes through targeted mutagenesis of *FLOURY ENDOSPERM 2* by using CRISPR/Cas9 approach and demonstrated that editing of *FLOURY ENDOSPERM 2* could be an effective route to reduce AC and accelerate rice breeding to achieve desired high ECQ trait. We have also analyzed the physicochemical properties of the *flo2* mutants, which have not only verified the improvements in rice ECQ, but also provided useful data for industrial evaluation of these potentially useful germplasm. Undesired, the mutations of *FLOURY ENDOSPERM 2* have led to a significant reduction in seed weight, which may imply a possible reduction in grain yield. This necessitates further evaluation for their agronomic performance, especially in the field conditions. Nevertheless, there might be a net gain in its output value given that the high value niche market for high-ECQ grains is rapidly expanding. Future studies could also be directed to the incorporation of high yielding traits, for example, by simultaneous targeting a number of negative regulatory genes, such as *GS3* (Zeng et al., 2019), *TGW6* (Hang et al., 2018; Zeng et al., 2019) and *GS6* (Sun et al., 2013), leading to an optimal balance between quality and yield traits.

## Materials and methods

### Plant materials and growth conditions

Suken118 (SK118) is an elite japonica rice variety with good resistance and quality, which is cultivated widely in the central of Jiangsu Province, China (http://ricedata.cn). The 20bp long target sites were chosen in the *FLOURY ENDOSPERM 2* codon region. The CRISPR/Cas9 vector was constructed as previously described then the cassette was transferred into SK118 callus by Agrobacterium tumefaciens-mediated transformation using the strain EHA105 (Hiei et al., 1997; Ma et al., 2015). Transgenic rice lines were grown in greenhouse and test fields in Jiangsu Academy of Agricultural Sciences in Nanjing, China, during normal rice-growing seasons.

### Genotype assays and phenotype

Positive T0 transgenic plants were identified by PCR amplification of a fragment of the Hyg gene that was used as a selection marker. The target genomic region of *FLOURY ENDOSPERM 2* was amplified by a pair of primers (*FLO2*-TF/TR; **Table S1**) flanking the target site and sequenced. Seeds were collected for each plant from WT and homozygous mutant lines. Grain length, plant height, the panicle length, the number of grains per panicle and tiller numbers per plant were measured in the fields. The 1,000 grains weight was measured after the oven at 45°C until constant weight. The grain length, width, and thickness were measured using a vernier caliper.

### Grain phenotype and iodine staining of endosperm

The hulls of rice seeds were removed to observe the external appearance of the grain in both WT and mutant lines. Grains were cut through the center to expose the endosperm; 1μL 0.1% iodine-potassium iodide solution was dropped on the endosperm surface and photographs were taken after 3-5 min by VHX-500FE.

### RNA isolation and qPCR

Total RNAs were extracted from the rice endosperm of rice using a TRIzol kit according to the user’s manual (Invitrogen) for expression analysis. And then reverse-transcribed into cDNA from 1 μg of total RNA following the manufacturer’s instructions (Fastking RT Kit; TIANGEN). qRT-PCR analysis was performed using a Light Cycler 480 system (Roche) and SYBR Green Real-time PCR Master Mix. The relative expression levels of *FLOURY ENDOSPERM 2* were normalized to the rice *UBIQUITIN* (Os03g0234200) gene, which was used as an internal control. All date were measured by three individual replicates. The transcript levels were calculated by the 2^-ΔΔCT^ method. The primer sequences are listed in **Table S1**.

### Determination of AC and total protein content

Amylose content (AC) was measured using an AA3 continuous flow analyzer set. Flour from WT, mutant lines and four rice samples with known apparent amylose content was taken 0.05 g to determine. AA3 continuous flow analyzer set was also used to determine total protein content by measuring 0.2 g of flour from WT and mutant lines. The conversion coefficient is 5.95. All samples were repeated three times.

### Evaluation of grain GC, GT and viscosity property

Gel consistency (GC) was measured following the procedure described in GB/T 22294-2008/ISO 11747:2012. Gelatinization temperature (GT) was indirectly estimated *via* the alkali digestion test. Rice viscosity properties were determined using a Rapid Visco Analyser (RVA-TecMaster, Newport Scientific, Warriewood, Australia). Rice flour (3g, 12% m.b.) was mixed with 25 g of double-deionized water in the RVA sample can. Thrice measurements were performed for each sample.

### Scanning electron microscopy of seed cross-section

After drying, the mature seeds with shelling were frozen in liquid nitrogen for 5S then the cross-section of the samples was manually snapped and sputter-coated with gold palladium on copper studs. Magnifications of about 25×, 500×, and 1000× were used to observe endosperm cross-section and starch granule morphology.

## Data availability statement

The original contributions presented in the study are included in the article/**Supplementary Material**, further inquiries can be directed to the corresponding authors.

## Author contributions

JY, GL and YF designed and supervised the research. XS, ZC, XD, FW and JW performed most experiments. YX and WL analyzed date. XS, BL, YT and YL wrote the paper. X-LT and YZ provided resources. All authors contributed to the article and approved the submitted version.
