# Supplementary material for: Generation of new rice germplasms with low amylose content by CRISPR/CAS9-targeted mutagenesis of the FLOURY ENDOSPERM 2 gene
Source: Front Plant Sci. 2023 Mar 13;14:1138523. doi: 10.3389/fpls.2023.1138523 (PMC10040805; doi:10.3389/fpls.2023.1138523)
Supplement: Supplementary file 1 [file DataSheet_1.docx]

**SUPPLEMENTS**

Fig. S1. Expression analysis of *FLO2*.


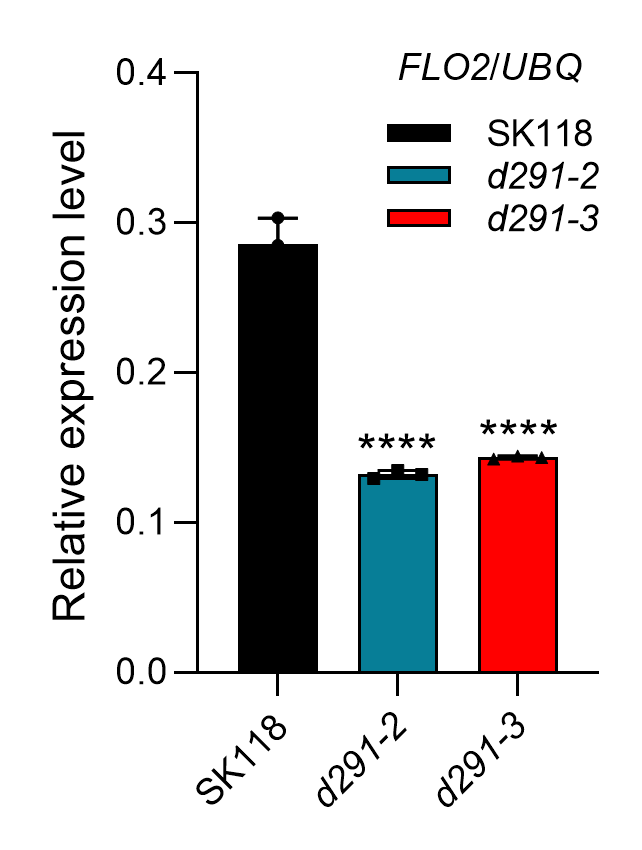


Fig. S1. Expression analysis of *FLO2*.

Rice *UBIQUITIN* was used as an internal control. Data are Mean ± SD from three individual replicates. Asterisks indicate statistical significance as determined by Student’s t-test (**, P < 0.01).

Table S1. Primers used in this study.

| **Primers name** | **Primer sequence（5’-3’）** |
| --- | --- |
| U3-gRNA-F | GGCATGCCGTCAACTTCGGAACAA |
| U3-gRNA-R | AAACTTGTTCCGAAGTTGACGGCA |
| U-F | CTCCGTTTTACCTGTGGAATCG |
| U-R | CGGAGGAAAATTCCATCCAC |
| Uctcg-B1’ | TTCAGAGGTCTCTCTCGCACTGGAATCGGCAGCAAAGG |
| gRcggt-BL | AGCGTGGGTCTCGACCGGGTCCATCCACTCCAAGCTC |
| SP1 | CCCGACATAGATGCAATAACTTC |
| SP2 | GCGCGGTGTCATCTATGTTA |
| *Hyg*-F | TCCGGAAGTGCTTGACATT |
| *Hyg*-R | GTCGTCCATCACAGTTTGC |
| Cas9-F | AGCGGCAAGACTATCCTCGACT |
| Cas9-R | TCAATCCTCTTCATGCGCTCCC |
| *FLO2*-TF | TTTGATCTGCTTTGGCCTTG |
| *FLO2*-TR | AGATAAATCCCCGTAACTCT |
| q*FLO2*-F | CACACCCTCCAGCAATATCA |
| q*FLO2*-R | CCTTCTGCGACTGCTTTTCT |
| q*UBQ*-F | GCCCAAGAAGAAGATCAAGAAC |
| q*UBQ*-R | CATATACCACGACCGTCAAAAC |
